# Taming a monomeric [Cu(η^6^-C_6_H_6_)]^+^ complex with silylene†
†Electronic supplementary information (ESI) available: Experimental details of **2**, **3**, **5** and **6**, their single crystal X-ray data, and details of theoretical calculations. CCDC 1540046, 1540047, 1547791, and 1547792. For ESI and crystallographic data in CIF or other electronic format see DOI: 10.1039/c8sc00459e
‡
‡Dedicated to Prof. Krishna N. Ganesh on the occasion of his 65th birthday.


**DOI:** 10.1039/c8sc00459e

**Published:** 2018-04-13

**Authors:** Nasrina Parvin, Shiv Pal, Jorge Echeverría, Santiago Alvarez, Shabana Khan

**Affiliations:** a Department of Chemistry , Indian Institute of Science Education and Research Pune , Dr Homi Bhaba Road, Pashan , Pune-411008 , India . Email: shabana@iiserpune.ac.in; b Inorganic Chemistry Department , Facultat de Química , Universitat de Barcelona , Diagonal, 645 , 08028 Barcelona , Spain . Email: jorge.echeverria@qi.ub.es ; Email: santiago.alvarez@qi.ub.es

## Abstract

Previous theoretical and experimental endeavors suggested that [Cu(C_6_H_6_)]^+^ prefers the η^1^/η^2^ mode over the η^6^ mode due to the augmented repulsion between the benzene ring and metal d-electrons. Nevertheless, the use of silylene as a neutral ligand has led to the isolation of the first monomeric copper cation, [{PhC(N*t*Bu)_2_SiN(SiMe_3_)_2_}Cu(η^6^-C_6_H_6_)]^+^[SbF_6_]^–^ (**3**), where a copper atom is bound to the benzene ring in an unsupported η^6^ fashion. However, the use of IPr (1,3-bis(2,6-diisopropylphenyl)imidazol-2-ylidene) in place of silylene results in the formation of [IPr·Cu(η^2^-C_6_H_6_)]^+^[SbF_6_]^–^ (**6**), where the copper atom is bound to the benzene ring in the η^2^ mode. The discrepancy in hapticities is also reflected when hexamethylbenzene is employed as the arene ring. The silylene supported copper cation continues to bind in the η^6^ mode in **2** while the NHC copper cation displays an η^3^ bonding mode in **5**. DFT calculations are carried out to understand how the use of silylene led to the η^6^ binding mode and why IPr afforded the η^2^ binding mode.

## Abstract 2

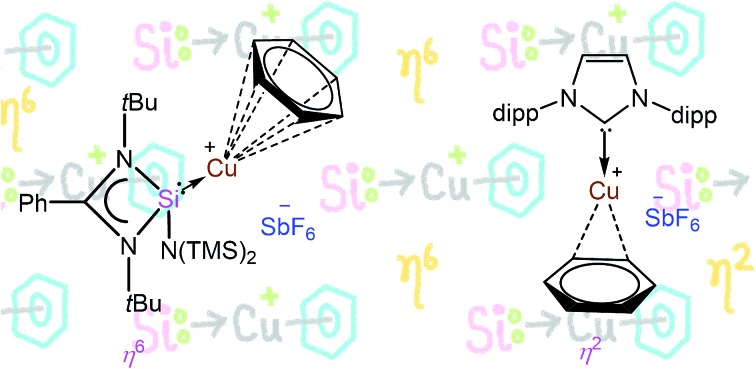
Realization of a hitherto elusive unsupported η^6^ binding mode of benzene to a copper(i) cation employing silylene as a ligand. The back-donation from Cu to Si(ii) diminishes the repulsion between d-electrons and the benzene ring and enforces the η^6^ binding mode.

## Introduction

Synthetic chemists often find fascination in isolating a compound that has been theoretically predicted as well as observed in the gas phase but never realized under laboratory conditions. However, access to such compounds often poses a formidable synthetic challenge. One such moiety is [Cu(η^6^-C_6_H_6_)]^+^. It is well evident from the literature that group 11 metal–arene complexes strongly prefer the η^2^ binding mode.^1^,^2^ Armentrout and coworkers reasoned that the preference of the η^2^ bonding mode over η^6^ is due to the increase of repulsion between the metal d-electrons and the benzene ligand in the latter.^3^ Cu–arene complexes with the η^6^ bonding mode have also been reported albeit in small numbers, when using tethered arene rings in order to create a cavity between the two arene rings by diminishing the repulsion.^4^ These experimental results were further computationally supported by Guo and co-workers, who found that in the gas phase free Cu^+^ may form η^6^ type of complexation with benzene but in the condensed phase the propensity of Cu^+^ to form η^2^ complexes with benzene drastically increases in the presence of a counter-anion.^5^

A major breakthrough in this research was recently achieved by Hayton and coworkers, who isolated two half sandwich complexes [(η^6^-C_6_Me_6_)Cu(PR_3_)][PF_6_] (R = Ph, OPh) where the C_6_Me_6_ ring is bound to the Cu ion in the η^6^ coordination mode.^6^ However, they have also theoretically shown that when benzene is employed instead of hexamethylbenzene as an arene, the η^2^ mode is preferred, and hence surmised that the preference for the η^6^ mode over the η^2^ mode is exclusively due to steric repulsion between Me groups and PR_3_ units. Additionally, they calculated the relative energies for [Cu(C_6_H_6_)]^+^ in gas as well as condensed phases and found the preference for the η^2^ mode in both phases but more in the condensed phase, as previously predicted by Guo *et al.* These studies consequently lead to the question: is it even possible to isolate [Cu(η^6^-C_6_H_6_)]^+^ in the condensed phase?

It is apparent now that one of the main factors responsible for the success or failure of the synthesis of [Cu(η^6^-arene)]^+^ is the ligand with an appropriate substituent. Being better σ-donors than phosphines, silylenes have recently been found to attract widespread interest as ligands for transition metals.^7^ For this challenging work, we turned our attention towards a [PhC(N*t*Bu)_2_SiN(SiMe_3_)_2_]^8^ supported copper bromide complex, [{PhC(N*t*Bu)_2_}Si{N(SiMe_3_)_2_}]_2_Cu_2_Br_2_ (**1**).^9^ An appealing facet of [PhC(N*t*Bu)_2_SiN(SiMe_3_)_2_] is that it accepts electron density from the metal as evidenced in its coinage metal complexes.^9^,^10^ We postulate that such back-donation can diminish the electrostatic repulsion between metal d-electrons and arene rings, which may facilitate the formation of the η^6^ mode. This potential has been duly realized through the isolation and characterization of an unprecedented [Cu(η^6^-C_6_H_6_)]^+^ complex. For a direct systematic comparison, we carried out the same reactions with N-heterocyclic carbene in place of **1**. Our results are reported herein.

## Results and discussion

**Scheme 1:**
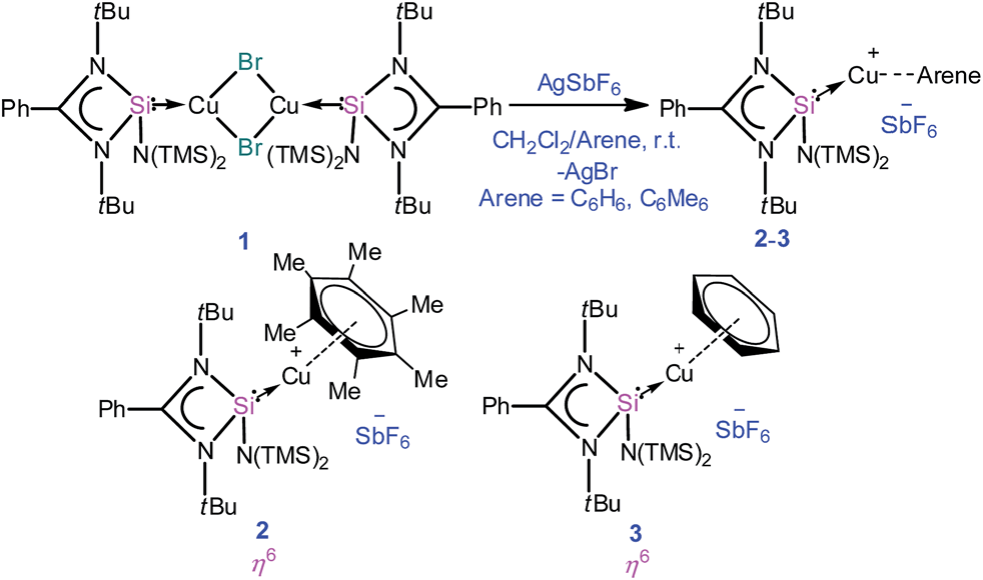
Synthesis of complexes **2** and **3**.

A simple synthetic protocol was designed to generate the desired copper cations. To check the credentials of **1** as a ligand, we commenced our investigation by probing the reaction of **1** with AgSbF_6_ in the presence of hexamethylbenzene with the assumption that it would furnish [(η^6^-C_6_Me_6_)Cu]^+^ analogous to Hayton's results. Gratifyingly, the abstraction of bromide ions from a dichloromethane solution of **1** with AgSbF_6_ in the presence of hexamethylbenzene results in the formation of [{PhC(N*t*Bu)_2_SiN(SiMe_3_)_2_}Cu(η^6^-C_6_Me_6_)]^+^[SbF_6_]^–^ (**2**) (see S1 in the ESI for experimental details†) (Scheme 1).

**Fig. 1:**
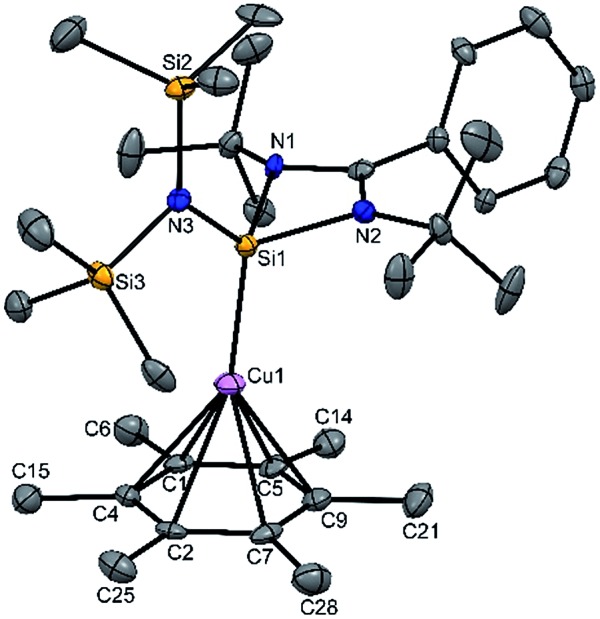
The molecular structure of **2** (ellipsoids are shown at the probability level of 50%). Counter anion SbF_6_^–^ and hydrogen atoms are omitted for clarity. Selected bond lengths (Å): Si1–Cu1 2.219(1), Si1–N1 1.837(3), Si1–N2 1.834(3), Cu1–C1 2.449(5), Cu1–C2 2.372(4), Cu1–C4 2.443(5), Cu1–C5 2.407(4), Cu1–C7 2.318(4), Cu1–C9 2.310(4), C1–C4 1.408(6), C4–C2 1.414(5), C2–C7 1.420(6), C7–C9 1.410(6), C9–C5 1.409(6), and C5–C1 1.414(6).

The molecular structure of **2** is shown in Fig. 1, which revealed the η^6^ mode of the arene ring. The Si atom adopts a distorted tetrahedral geometry with a Si→Cu bond length of 2.219(1) Å, which is in good accordance with that in **1** [2.222(2) Å].^10^ The Cu–C(arene) bond lengths varies from 2.310(4) to 2.449(5) Å, reflecting a slightly unsymmetrical binding of the Cu with respect to the ring. The C–C bond lengths in the arene ring are more or less the same ranging from 1.408(6) to 1.420(6) Å. The distance between the Cu atom and the centroid of the arene ring is 1.920 Å.

**Fig. 2:**
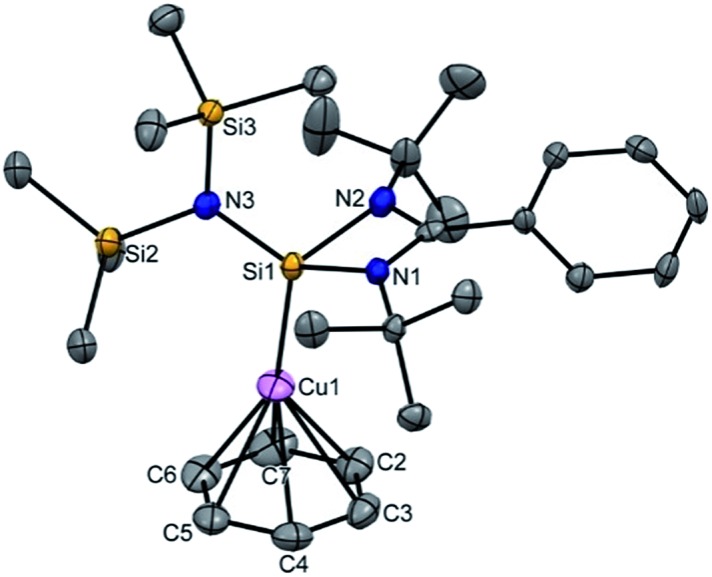
The molecular structure of **3** (ellipsoids are shown at the probability level of 50%). Counter anion SbF_6_^–^ and hydrogen atoms are omitted for clarity. Selected bond lengths (Å): N2–Si1 1.844(4), N1–Si1 1.847(5), N3–Si1 1.737(5), Cu1–Si1 2.231(2), Cu1–C2 2.454(7), Cu1–C3 2.477(8), Cu1–C4 2.413(9), Cu1–C5 2.359(9), Cu1–C6 2.342(9), Cu1–C7 2.379(8), C2–C3 1.38(1), C3–C4 1.39(1), C4–C5 1.39(1), C5–C6 1.39(1), C6–C7 1.40(1), C7–C2 1.40(1), and Cu1–centroid of the benzene ring 1.960.

Next, we turned our endeavours towards our primary objective of isolating [Cu(η^6^-C_6_H_6_)]^+^. A similar synthetic protocol was adopted to access [{PhC(N*t*Bu)_2_SiN(SiMe_3_)_2_}Cu(η^6^-C_6_H_6_)]^+^[SbF_6_]^–^ (**3**). Complex **3** crystallizes in the monoclinic space group *P*2_1_/*n*. The molecular structure of **3** (Fig. 2) reveals the η^6^ coordination mode of benzene to the Cu center. The Cu–C_benzene_ bond distances range from 2.342(9) to 2.477(8) Å, with an average of 2.404 Å, which is longer than those reported for [Cu(η^6^-C_6_Me_6_)]^+^.^6^ Similarly, the distance between the Cu atom and the centroid of the benzene ring (Cu–C_centroid_ 1.960 Å) in **3** is slightly longer than those in Hayton's [Cu(η^6^-C_6_Me_6_)]^+^ complexes (1.800(3) and 1.775(6) Å),^6^ but significantly shorter than those reported for the tethered Cu(arene) complexes such as Cu(i)-cyclophanes or 9,10-anthracene derived endo-cyclic Cu(i) complexes (∼2.5–3.0 Å).^4^ The [SbF_6_] anion in the asymmetric unit shows no significant bonding interaction with the Cu^+^ atom and the closest approach between the F atom and the Cu center (Cu···F) is 4.96(1) Å, which rules out any possibility of interaction between them. The average C–C bond length of the C_6_H_6_ ligand in **3** is 1.39 Å (range 1.38(1)–1.40(1) Å) (C–C_C_6_H_6_(non-bound)_: 1.40 Å; C–C_Me_6_C_6_(non-bound)_: 1.41 Å). The Si(ii) atom assumes a distorted tetrahedral geometry with a Si(ii)→Cu bond length of 2.231(2) Å, which is similar to that in **1** and **2**.

**Scheme 2:**
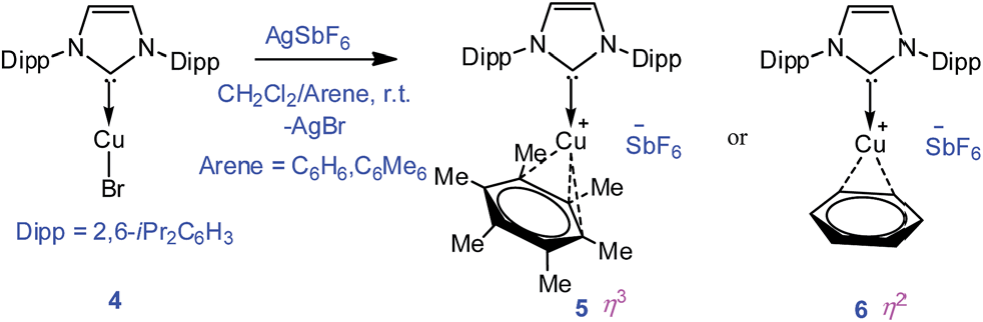
Synthesis of complexes **5** and **6**.

All analytical and spectroscopic data of **2** and **3** are consistent with the proposed structures. The binding of benzene to the Cu atom in **3** resulted in a slight downfield shift of the C_6_*H*_6_ protons (*δ* 7.46 ppm). The appearance of two signals for the trimethylsilyl groups in ^1^H (*δ* 0.24 and 0.39 ppm) as well as ^29^Si NMR (*δ* 7.21 and 7.65 ppm) of **3** indicates that they are not equivalent and the diastereotopicity arises from the bulky substituents around the Si(ii) atom. The Si(ii) center resonates at *δ* 4.41 ppm, which is marginally upfield relative to that in **1** (*δ* 5.72 ppm) in the ^29^Si NMR spectrum (Scheme 2).^10^

**Fig. 3:**
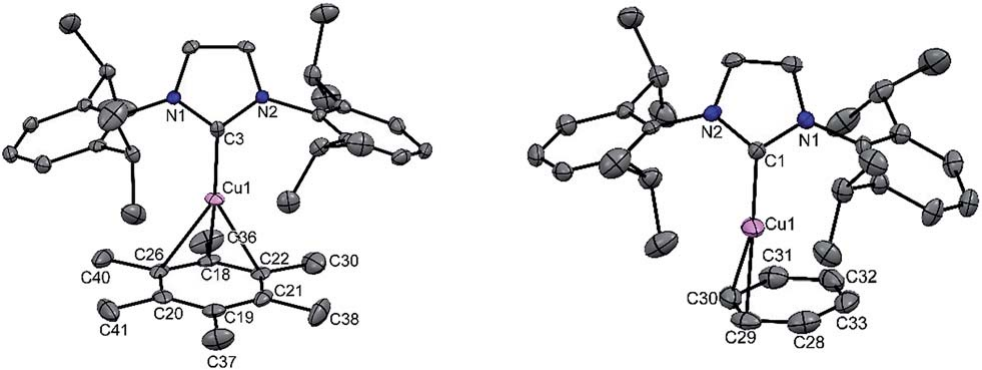
Molecular structure of **5** and **6** (ellipsoids are shown at the probability level of 50%). Counter anion SbF_6_^–^ and hydrogen atoms are omitted for clarity. Selected bond lengths (Å) **5**: N1–C3 1.353(4), N2–C3 1.356(4), Cu1–C3 1.890(3), Cu1–C18 2.114(4), Cu1–C22 2.289(4), Cu1–C26 2.319(4), Cu1–C21 2.678(4), Cu1–C20 2.715(4), and Cu1–C19 2.894(4). **6**: C1–N1 1.356(7), C1–N2 1.349(6), C1–Cu1 1.886(5), Cu1–C29 2.129(6), Cu1–C30 2.217(5), Cu1–C28 2.456(6), Cu1–C31 2.621(5), Cu1–C33 2.813(6), and Cu1–C32 2.892(6).

To extend the analogous chemistry with N-heterocyclic carbenes we reacted the previously reported IPr·CuBr (**4**)^11^ with AgSbF_6_ in the presence of hexamethylbenzene and benzene, which afforded [IPr·Cu(η^3^-C_6_Me_6_)]^+^[SbF_6_]^–^ (**5**) and [IPr·Cu(η^2^-C_6_H_6_)]^+^[SbF_6_]^–^ (**6**), respectively. Single crystal X-ray studies on **5** and **6** indicated η^3^ and η^2^ coordination^12^ of the Cu atom with the arene rings, respectively (Fig. 3) (please see S3 in the ESI† for the deduction of hapticities in **5** and **6**). The C_IPr_–Cu bond lengths in **5** and **6** are 1.890(3) and 1.886(5) Å, respectively. The Cu–C_arene_ bond lengths in **5** range from 2.114(4) to 2.319(4) Å, and from 2.129(6) to 2.217(5) Å for **6**. The methyl protons of the C_6_Me_6_ ring appear at *δ* 1.8 ppm with an integration of 18 protons.

In order to understand the different hapticities observed experimentally, the geometries of **2**, **3**, **5**, and **6** have been optimized at the DFT level (see the ESI† for a detailed description of the computational procedure and for the atomic coordinates of the optimized structures).

The hapticities and the most relevant bond distances, calculated at the B3LYP-D3 level, are shown in Table 1, together with the experimental values. It can be seen that the experimental Cu–L (L = Si, C) distances are reproduced within 0.02 Å and the Cu–arene ones within 0.1 Å. The η^6^ coordination in the silylene complexes is well reproduced by our calculations. The hapticities for other cases for which η^1^, η^2^ and η^3^ coordinations can be hard to distinguish are deduced from the values of the distance ratios of the three shortest Cu–C_arene_ distances (*d*_1_ < *d*_2_ < *d*_3_), *ρ*_1_ (*d*_2_/*d*_1_) and *ρ*_2_ (*d*_3_/*d*_1_).^12^ For the carbene complexes, the calculated hapticity is η^3^ for **5** and η^2^ for **6**, as indicated by the corresponding values of *ρ*_1_ and *ρ*_2_. It is worth mentioning here that carbene complexes evolve to η^3^/η^2^ when starting the optimization from an η^3^ geometry, whereas silylene complexes behave conversely.

**Table 1:** Optimized geometrical parameters of the four molecules under study. The dispersion-corrected B3LYP-D3 method and the 6-31G* basis set for N and Cu and 6-311+G* for H and C were employed. The numbers given in parentheses are the Cu–arene distance ratios *ρ*_1_ and *ρ*_2_

Molecule	Hapticity (*ρ*_1_, *ρ*_2_)	Cu–L (Å)	Shortest Cu–C_arene_ (Å)
**2**	Exp. η^6^	2.219	2.310
	Calcd. η^6^	2.206	2.298
**3**	Exp. η^6^	2.231	2.342
	Calcd. η^6^	2.209	2.272
**5**	Exp. η^3^(1.05, 1.09)	1.890	2.114
	Calcd. η^3^(1.08, 1.13)	1.886	2.078
**6**	Exp. η^2^ (1.04, 1.15)	1.886	2.129
	Calcd. η^2^ (1.01, 1.33)	1.887	2.105

**Scheme 3:**
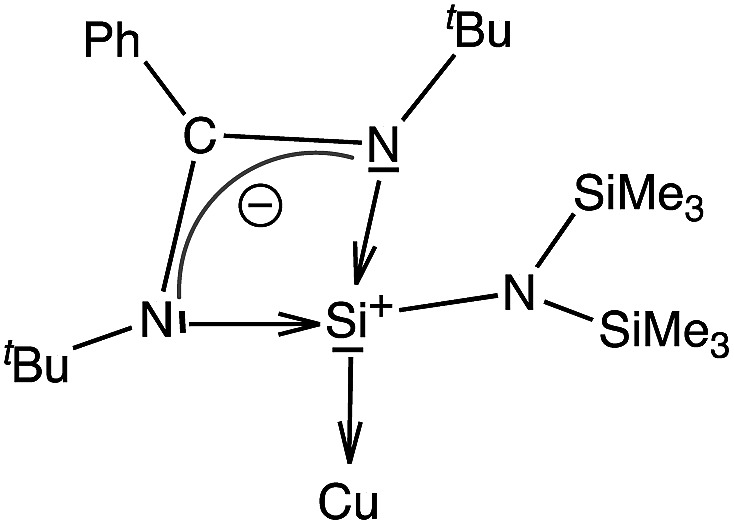
Zwitterionic form of the silylene ligand.

We have performed an NBO analysis of the benzene complexes **3** and **6** to try to rationalize their different behavior in terms of arene coordination. Second order perturbation analysis revealed that bonding between Cu and the carbene ligand is a donor–acceptor interaction from the carbene lone pair to an empty Cu orbital (nC→nCu*, *E* = 109.1 kcal mol^–1^). Cu–silylene donor–acceptor interactions in **3**, however, are not clearly determined because the complex could not be decomposed into the same fragments as in **6**. On the other hand, the coordination of the benzene ring to the metal atom is associated with donor–acceptor interactions involving a mixture of s and p benzene orbitals (98 and 84 kcal mol^–1^ in **3** and **6**, respectively). Moreover, for the carbene complex there is π-back donation towards the benzene ring (nCu→πC–C*, *E* = 19.3 kcal mol^–1^). Another relevant result is that the atomic charge on the donor atoms is –0.06 for C_IPr_ in **6**, but +1.26 for the Si atom in **3**. These values are consistent with the zero-valent nature of the carbenoid carbon atom and the formal positive charge of the Si atom in the zwitterionic Lewis structure of the ligand (Scheme 3), calculated to be +1.18 for the free ligand. The calculated charge on Si in **3** is thus the result of a formal positive charge increased by σ donation, partially compensated by π back-donation from Cu.

The presence of H···H attractive interactions,^13^ involving the arene's hydrogen atoms on one side, and those of the ^i^Pr and SiMe_3_ groups of the carbene and silylene ligands on the other side, might also have some effect on the different stabilities of the η^6^ coordination in the two cases. The optimized complexes present numerous dihydrogen contacts between the arene and the silylene ligand at distances in the range of 2.3–2.5 Å (consistent with C···C distances of 3.5–3.9 Å in the crystal structures). Such intramolecular interactions have been shown to stabilize otherwise unstable systems, as for example in the case of molecules with very long C–C bonds^14^ or the *cis* form of a substituted azobenzene.^15^

We have performed NCI (non-covalent interaction) calculations^16^ (see the NCI maps of **2**, **3**, **5** and **6** in ESI, S4†), observing regions of attractive non-covalent interactions between the hydrogen atoms of the benzene and the methyl groups of the silylene in **3** (also in the hexamethylbenzene complex **2**) and between the benzene hydrogens and the carbene ^i^Pr groups in **6**. Indeed, AIM analysis of **3** discloses a bond path between the H atoms of the coordinated C_2_H_2_ moiety of benzene and those of the ^i^Pr groups of the carbene, with an electron density at a bond critical point of 0.003 au, similar to previously reported dihydrogen interactions.^17^

To further test the relative influence of steric repulsions and non-covalent interactions on the hapticity of the coordinated arenes we have carried out energy decomposition analysis (EDA) for **3** and **6** (Table 2), as well as for the hypothetical complex **6′** in which the benzene is forced to be coordinated in an η^6^ mode. In this constrained model complex, which is not a minimum of the potential energy surface, the Cu–C_arene_ distances were set to those of **3**. In general, the interaction energy and its decomposition was evaluated between two molecular fragments: the C_6_H_6_ ring and the Cu(IPr) and Cu(silylene) fragments, respectively. It can be seen that the results for **6′** indicate a much stronger Pauli repulsion between the two ligands than in **3**, which is relieved by its slippage to an η^2^ coordination. Even if slippage also reduces the stabilizing components, the balance yields a more favorable interaction energy due to the dramatic decrease of the Pauli repulsion term. EDA analysis of the interaction between benzene and the complementary ligands in the absence of the Cu centre for the three compounds in Table 2 allows us to estimate a Pauli term that calibrates the part of the steric repulsion that comes from benzene–ligand interactions, and we can also roughly estimate the benzene–Cu repulsion as the difference between the total and the benzene–ligand Pauli terms. The important Pauli repulsion between the η^6^-C_6_H_6_ molecule and the Cu ion can be attributed to the interaction between the occupied π(e_1g_) orbitals of benzene and the d_*xz*_ and d_*yz*_ orbitals of Cu. Hence, the smaller repulsion in η^6^-**3** compared to η^6^-**6′** must also be attributed to the longer Cu–Si/C_carbene_ distance in the former case (2.21 Å in **3***vs.* 1.89 Å in **6′**). Clearly, slippage of the benzene ring from the η^6^ coordination in **6′** to the η^2^ mode in **6** results in a significant decrease in both the benzene–carbene and benzene–copper Pauli repulsions, and explains the preference for the η^2^ mode in the latter, in contrast with the preference for the η^6^ coordination in **3**.

**Table 2:** Energy decomposition analysis (EDA) of electrostatic (Δ*E*_elect_), dispersion (Δ*E*_disp_), polarization (Δ*E*_pol_), charge transfer (Δ*E*_CT_) and Pauli repulsion (Δ*E*_Pauli_) terms for compounds **3**, **6** and **6′**, corrected for the BSSE. The interaction is defined between the C_6_H_6_ ring and the Cu(IPr) and Cu(silylene) fragments, respectively; energies are given in kcal mol^–1^

Cpd.	Δ*E*_int_	Δ*E*_elect_	Δ*E*_disp_	Δ*E*_pol_	Δ*E*_CT_	Δ*E*_Pauli_		
						Total	C_6_H_6_·L	C_6_H_6_·Cu
**3** (η^6^)	–23.3	–51.7	–15.4	–25.0	–27.5	96.4	10.6	85.8
**6′** (η^6^)	–17.4	–70.6	–22.0	–27.0	–32.8	134.9	37.5	97.4
**6** (η^2^)	–38.9	–36.4	–15.0	–19.7	–23.0	55.4	7.2	48.2

The dispersion interaction contributes significantly to the bonding between the C_6_H_6_ ring and the CuL fragments (L = {PhC(N*t*Bu)_2_SiN(SiMe_3_)_2_} and IPr for **3** and **6**, respectively), and outweighs in both cases the benzene–ligand steric repulsions, contributing some 5–8 kcal mol^–1^ to the bonding between the benzene and the CuL fragment. It must be noted that the dispersion contribution is similar in the two cases and therefore has a negligible effect on the hapticity.

## Conclusions

This study was undertaken to synthesize the first copper cation bound to the benzene ring in an unsupported η^6^ mode. The silylene supported copper cation was found to be bound with both benzene and hexamethylbenzene in an η^6^ mode. DFT calculations revealed that the positive charge on silylene favors back-donation from the Cu atom, thus relieving the repulsions between the benzene π-system and the Cu d-electrons. Furthermore, the long Cu–Si bond distance places the *t*Bu substituents of the silylene at a longer distance from the arene hydrogens, thereby significantly reducing the steric repulsion that prevents the η^6^ coordination in the case of the NHC complexes. We conclude that, to favor an η^6^ coordination mode, the complementary ligands must have a π-acceptor character, with a third row donor atom to minimize steric repulsions, and with a relatively small cone angle. Based on these principles, we have carried out test calculations on the so far unprepared [(C_6_H_6_)Cu(CN)] and [(C_6_H_6_)Cu(CO)]^+^ complexes that disclose an η^6^ coordination in both cases and similar bonding parameters to those in compound **3** reported herein.

## Conflicts of interest

There are no conflicts to declare.

## Supplementary Material

Supplementary information

Crystal structure data
