# Supplementary material for: Taming a monomeric [Cu(η6-C6H6)]+ complex with silylene
Source: Chem Sci. 2018 Apr 13;9(18):4333–7. doi: 10.1039/c8sc00459e (PMC5944381; doi:10.1039/c8sc00459e)
Supplement: Supplementary file 1 [file SC-009-C8SC00459E-s001.pdf]

Electronic Supplementary Material (ESI) for Chemical Science.
This journal is © The Royal Society of Chemistry 2018

# Taming Monomeric $[Cu(\eta^6\text{-}C_6H_6)]^+$ Complex with Silylene

Nasrina Parvin,[a] Shiv Pal,[a] Jorge Echeverría,*[b] Santiago Alvarez,*[b] and Shabana Khan*[a]

*[a] Department of Chemistry*

*Indian Institute of Science Education and Research Pune*

*Dr. Homi Bhaba Road, Pashan, Pune - 411008, India*

*E-mail: shabana@iiserpune.ac.in*

*[b] Inorganic Chemistry Department,*

*Facultat de Química,*

*Universitat de Barcelona, Diagonal, 645, 08028*

*Barcelona, Spain.*

## Content:

S1. Experimental section

S2. Crystal data and structure refinements for **2**, **3**, **5**, and **6**

S3. Deduction of hapticities in **5** and **6**

S4. NCI plot of **2**, **3**, **5**, and **6**

S5. Cartesian coordinated of optimized structures **2**, **3**, **5**, and **6**

S6. Computational details

S7. References

**S1. Experimental Section:**

All experiments were carried out under an atmosphere of dry argon or in vaccuo using standard Schlenk technique and in a dinitrogen filled MBRAUN MB 150-G1 glovebox. The solvents used were purified by MBRAUN solvent purification system MB SPS-800. The starting materials **1** and **4** were prepared as reported in the literature.[1] All other chemicals purchased from Aldrich were used without further purification. $^{1}H$, $^{13}C$, $^{19}F$ and $^{29}Si$ NMR spectra were recorded with Bruker 400 MHz spectrometer, using $CDCl_3$ and $CD_2Cl_2$ as solvent with an external standard ($SiMe_4$ for $^{1}H$, $^{13}C$ and $^{29}Si$ and trifluorotoluene for $^{19}F$). Mass spectra were recorded using AB Sciex, 4800 plus MALDI TOF/TOF.

**Synthesis of 2:**

30 mL $CH_2Cl_2$ was added to a 100 mL Schlenk flask containing mixture of **1** (0.281g, 0.25 mmol), $AgSbF_6$ (0.171g, 0.5 mmol) and $C_6Me_6$ (0.081 g, 0.5 mmol). After overnight stirring, AgBr was separated out from the reaction mixture by filtration and colorless block shaped crystals were observed in $CH_2Cl_2$-pentane mixture at 0 $^{o}C$. Yield: 0.280g (64%). Mp: 130 $^{o}C$ (decomposition). $^{1}H$ NMR (400 MHz, $CD_2Cl_2$, 298K): $\delta$ 0.20 (*s*, 9H, Si*Me₃*), 0.27 (*s*, 9H, Si*Me₃*), 1.06 (*s*, 18H, C*Me₃*), 2.34 (*s*, 18H, $C_6$*Me₆*), 7.31-7.36 (*m*, 1H, Ph), 7.42-7.56 (*m*, 4H, Ph) ppm. $^{13}C\{^{1}H\}$ NMR (100.613 MHz, $CD_2Cl_2$, 298 K): $\delta$ 4.59 ($SiMe_3$), 5.15 ($SiMe_3$), 17.59 ($C_6Me_6$), 31.54 ($CMe_3$), 54.61 ($CMe_3$), 126.11, 127.92, 128.47, 128.87, 129.89, 130.56, 131.32 (Ph-*C*), ppm. $^{29}Si\{^{1}H\}$ NMR (79.495 MHz, $CDCl_3$, 298 K): $\delta$ 7.83 ($SiMe_3$), 7.09 ($SiMe_3$), 0.02 ($SiN(SiMe_3)_2$). $^{19}F\{^{1}H\}$ NMR (376.49 MHz, $CDCl_3$, 298K): $\delta$ -176.70 (br) ppm. MALDI: *m/z* $[C_{33}H_{59}N_3Si_3Cu]^+$: 482.47 $[M-C_6Me_6]^+$. Anal Calcd: C, 44.97; H, 6.75; N, 4.77. Found: C, 44.89; H, 6.64; N, 4.82.

**Synthesis of 3:**

$AgSbF_6$ (0.171g, 0.5 mmol) was dissolved in $CH_2Cl_2$ (15 mL) and added to the solution of **1** (0.280g, 0.25 mmol) in benzene (15 mL). It was stirred overnight at room temperature. After that, AgBr was separated out from the reaction mixture by filtration and reduced the volume to 15 mL and kept it at 0 $^{o}C$. Colorless block shaped crystals suitable for X-ray analysis were observed after one day. Yield: 0.260 g (65 %). Mp: 112 $^{o}C$ (decomposition). $^{1}H$ NMR (400 MHz, $CDCl_3$, 298K): $\delta$ 0.24 (*s*, 9H, Si*Me₃*), 0.39 (*s*, 9H, Si*Me₃*), 1.15 (*s*, 18H, C*Me₃*), 7.22-7.32

(*m*, 1H, Ph), 7.33-7.43 (*m*, 2H, Ph), 7.46 (*s*, 6H, $C_6H_6$) 7.47-7.69 (*m*, 2H, Ph) ppm. $^{13}C\{^1H\}$ NMR (100.613 MHz, $CDCl_3$, 298 K): $\delta$ 3.51 (Si*Me₃*), 4.87 (Si*Me₃*), 30.72 (C*Me₃*), 53.91 (*C*Me₃), 126.55, 126.67, 126.92, 127.82, 129.23, 129.86, 130.08 (Ph-*C*), 168.91 (N*C*N) ppm. $^{29}Si\{^1H\}$ NMR (79.495 MHz, $CDCl_3$, 298 K): $\delta$ 7.65 (*Si*Me₃), 7.21 (*Si*Me₃), 4.41 (*Si*N(SiMe₃)₂). $^{19}F\{^1H\}$ NMR (376.49 MHz, $CDCl_3$, 298K): $\delta$ -173.64 (br) ppm. MALDI: *m/z* $[C_{27}H_{47}N_3Si_3Cu]^+$: 482.84 $[M-C_6H_6]^+$. Anal Calcd: C, 40.68; H, 5.94; N, 5.27. Found: C, 40.57; H, 6.05; N, 5.37.

**Synthesis of 5:**

30 mL $CH_2Cl_2$ was added to a 100 mL Schlenk flask containing mixture of **4** (0.266g, 0.5 mmol), $AgSbF_6$ (0.171g, 0.5 mmol) and $C_6Me_6$ (0.081 g, 0.5 mmol). After overnight stirring, AgBr was separated out from the reaction mixture by filtration and it was crystallized in saturated $CH_2Cl_2$ solution. Yield: 0.300g (70%). Mp: 175 °C (decomposition). $^1H$ NMR (400 MHz, $CDCl_3$, 298K): $\delta$ 1.10 (*dd*, 24H, *J*= 9.7, 6.9 Hz, $CH(CH_3)_2$), 1.80 (*s*, 18H, $C_6Me_6$), 2.20-2.30 (*m*, 4H, $CH(CH_3)_2$), 7.29 (*d*, 4H, *J*= 7.8 Hz, Ph), 7.52 (*t*, 2H, *J*= 7.8 Hz, Ph) ppm. $^{13}C\{^1H\}$ NMR (100.613 MHz, $CDCl_3$, 298 K): $\delta$ 16.21, 22.81, 23.19, 27.67, 123.34, 123.54, 128.27, 129.82, 133.62, 144.34, 175.90 ppm. $^{19}F\{^1H\}$ NMR (376.49 MHz, $CDCl_3$, 298K): $\delta$ -174.02 (br) ppm. MALDI: *m/z* $[C_{39}H_{54}N_2Cu]^+$: 613.43 $[M]^+$. Anal Calcd: C, 55.10; H, 6.40; N, 3.30. Found: C, 55.22; H, 6.60; N, 3.24.

**Synthesis of 6:**

$AgSbF_6$ (0.171g, 0.5 mmol) was dissolved in $CH_2Cl_2$ (15 mL) and added to the solution of **4** (0.266g, 0.5 mmol) in benzene (15 mL). After overnight stirring, AgBr was separated out from the reaction mixture by filtration and reduced the volume to 15 mL and kept it at 0°C. Colorless block shaped crystals suitable for X-ray analysis was observed after one day. Yield: 0.275g (72%). Mp: 228°C (decomposition). $^1H$ NMR (400 MHz, $CD_2Cl_2$, 298K): $\delta$ 1.14 (*dd*, 24H, *J*= 8.9, 7.0 Hz, $CH(CH_3)_2$), 2.25-2.32 (*m*, 4H, $CH(CH_3)_2$), 7.11 (*br*, 4H, $C_6H_6$), 7.18 (*s*, 2H, $C_6H_6$), 7.31 (*d*, 4H, *J*= 7.8 Hz, Ph), 7.53 (*t*, 2H, *J*= 7.8 Hz, Ph) ppm. $^{13}C\{^1H\}$ NMR (100.613 MHz, $CD_2Cl_2$, 298 K): $\delta$ 23.01, 23.68, 27.94, 123.62, 123.68, 130.25, 133.13, 144.74 ppm. $^{19}F\{^1H\}$ NMR (376.49 MHz, $CDCl_3$, 298K): $\delta$ -173.64 (br) ppm. MALDI: *m/z* $[C_{33}H_{42}N_2Cu]^+$: 451.19 $[M -C_6H_6]^+$. Anal Calcd: C, 51.74; H, 5.53; N, 3.66. Found: C, 51.62; H, 5.40; N, 3.57.

**S2. Crystal Data and Structure Refinements for 2, 3, 5, and 6:** Single crystals of suitable size, coated with paraffin oil was mounted for all the complexes. Crystal data for all the complexes were collected on a Bruker Smart Apex Duo diffractometer at 100 K using Mo Kα radiation (λ = 0.71073 Å). Collected data were integrated by using SAINT and then absorption correction was done by multi-scan method using SADABS program. All the structures were solved by direct methods and refined by full-matrix least-squares methods against $F^2$ (SHELXL-2014/6). CCDC No.: **2** (1540046), **3** (1540047), **5** (1547791) and **6** (1547792).

| | **2** | **3** | **5** | **6** |
|---|---|---|---|---|
| **Chemical formula** | $C_{36}H_{56}CuF_6N_3SbSi_3$ | $C_{33}H_{59}CuF_6N_3SbSi_3$ | $C_{40}H_{56}Cl_2CuF_6N_2Sb$ | $C_{33}H_{42}CuF_6N_2Sb$ |
| **Formula weight** | 914.39 | 881.39 | 935.08 | 766.00 |
| **Temperature** | 100(2) | 100(2) | 100(2) | 100(2) |
| **Wavelength** | 0.71073 | 0.71073 | 0.71073 | 0.71073 |
| **Crystal system** | monoclinic | monoclinic | monoclinic | monoclinic |
| **Space group** | $P\ 2_1/n$ | $P\ 2_1/c$ | $P\ 2_1/n$ | $P\ 2_1/c$ |
| $a$ | 14.617(7) Å | 13.605(4) Å | 10.506(5) Å | 13.3555(16) Å |
| $b$ | 14.759(7) Å | 14.057(4) Å | 20.813(9) Å | 13.9173(16) Å |
| $c$ | 21.243(10) Å | 21.790(8) Å | 20.439(9) Å | 18.181(2) Å |
| $\alpha$ | 90° | 90° | 90° | 90° |
| $\beta$ | 107.874(10)° | 107.833(9)° | 104.289(7)° | 102.245(3)° |
| $\gamma$ | 90° | 90° | 90° | 90° |
| **Volume** | 4361(4) Å$^3$ | 3967(2) Å$^3$ | 4176.4(18) Å$^3$ | 3302.4(7) Å$^3$ |
| ***Z*** | 4 | 4 | 4 | 4 |
| **Density (calculated)** | 1.393 g/cm$^3$ | 1.476 g/cm$^3$ | 1.487 g/cm$^3$ | 1.541 g/cm$^3$ |
| **Absorption coefficient** | 1.242 mm$^{-1}$ | 1.363 mm$^{-1}$ | 1.341 mm$^{-1}$ | 1.521 mm$^{-1}$ |
| **F(000)** | 1876 | 1816 | 1912 | 1552 |
| **Theta range for data collection** | 2.44 to 25.25° | 2.44 to 25.25° | 2.24 to 25.25 | 2.26 to 25.25° |
| **Index ranges** | -17<=h<=17<br>-17<=k<=17 | -16<=h<=16<br>-16<=k<=16 | -12<=h<=12<br>-24<=k<=24 | -16<=h<=16<br>-16<=k<=16 |

| | | | | |
|---|---|---|---|---|
| | -25<=l<=25 | -26<=l<=26 | -24<=l<=24 | -21<=l<=21 |
| **Reflections collected** | 77830 | 182758 | 142079 | 104497 |
| **Independent reflections** | 7893[R(int)=0.1631] | 7182[R(int)=0.1797] | 7557[R(int)=0.1114] | 5981[R(int)=0.1502] |
| **Coverage of independent reflections** | 99.9% | 99.9% | 99.9% | 99.9% |
| **Function minimized** | $\Sigma$ w(Fo$^2$ - Fc$^2$)$^2$ | $\Sigma$ w(Fo$^2$ - Fc$^2$)$^2$ | $\Sigma$ w(Fo$^2$ - Fc$^2$)$^2$ | $\Sigma$ w(Fo$^2$ - Fc$^2$)$^2$ |
| **Data/ restraints/ parameters** | 7893/ 18/ 451 | 7182/ 0/ 442 | 7557/ 0/ 483 | 5981/ 0/ 396 |
| **Goodness-of-fit on F2** | 1.009 | 1.094 | 1.034 | 1.027 |
| **Δ/ σ max** | 0.001 | 0.012 | 0.001 | 0.001 |
| **Final R indices** | 5006 data; [I>2σ(I)] *R*1= 0.0586, w*R*2= 0.1179 | 4904 data; [I>2σ(I)] *R*1= 0.0382, w*R*2= 0.0642 | 5835 data; [I>2σ(I)] *R*1= 0.0362, w*R*2= 0.0760 | 4129 data; [I>2σ(I)] *R*1= 0.0516, w*R*2= 0.0932 |
| | all data, *R*1= 0.1162, w*R*2= 0.1441 | all data, *R*1= 0.0834, w*R*2= 0.0772 | all data, *R*1= 0.0587, w*R*2= 0.0861 | all data, *R*1= 0.0958, w*R*2= 0.1086 |
| **Largest diff. peak and hole** | 1.218 and -0.860 eÅ$^{-3}$ | 0.817 and -0.657 eÅ$^{-3}$ | 1.309 and -0.601 eÅ$^{-3}$ | 1.001 and -0.937 eÅ$^{-3}$ |
| **R. M. S deviation from mean** | 0.127 eÅ$^{-3}$ | 0.111 eÅ$^{-3}$ | 0.091 eÅ$^{-3}$ | 0.108 eÅ$^{-3}$ |

## S3. Deduction of hapticities in 5 and 6

The Cu-C(arene) bond distance should be <2.50 Å to be considered for hapticity count. [*Angew. Chem., Int. Ed.,* 2015, **54**, 3088]

For **5**: Cu1-C21 2.678(4), Cu1-C20 2.715(4), Cu1-C19 2.894(4). As these bond distances are are >2.50 Å, they were not considered for hapticity count.

For **6**: Cu1-C28 2.456(6), Cu1-C31 2.621(5), Cu1-C33 2.813(6), Cu1-C32 2.892(6).

One Cu1-C28 2.456(6) distance is less than 2.50 Å, however, the difference from the coordinating Cu-C bond distances [Cu1-C29 2.129(6); Cu1-C30 2.217(5)] is quite large. Hence,

the hapticity count is difficult for such cases. We have therefore used the method given in *Organometallics,* 2014, **33**, 6660-6668.

It can be deduced from the values of the distance ratios of the three shortest Cu-$C_{arene}$ distances ($d_1 < d_2 < d_3$), $p_1$ ($d_2/d_1$) and $p_2$ ($d_3/d_1$)

η1 coordination: $\rho1 \approx \rho2 \gg 1$

η3 coordination, (we expect the three M−C bond distances to be approximately equivalent) $\rho1 \approx \rho2 \approx 1$

η2 coordination, for which $d1 \approx d2 < d3$, $\rho2 > \rho1 \approx 1$.

**S4**. **NCI plots** of **2**, **3**, **5** and **6**.

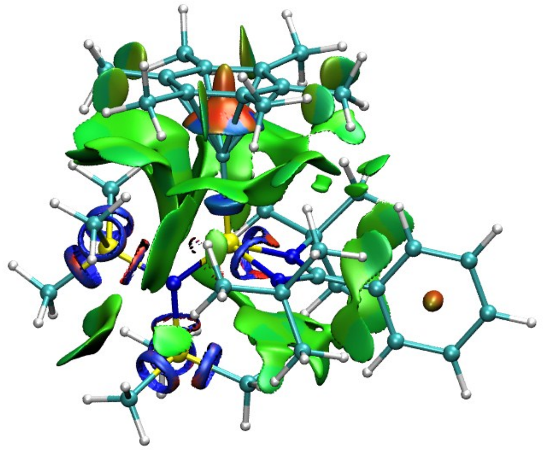

**2**

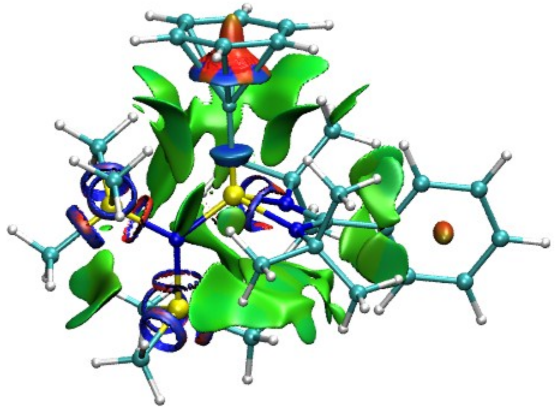

**3**

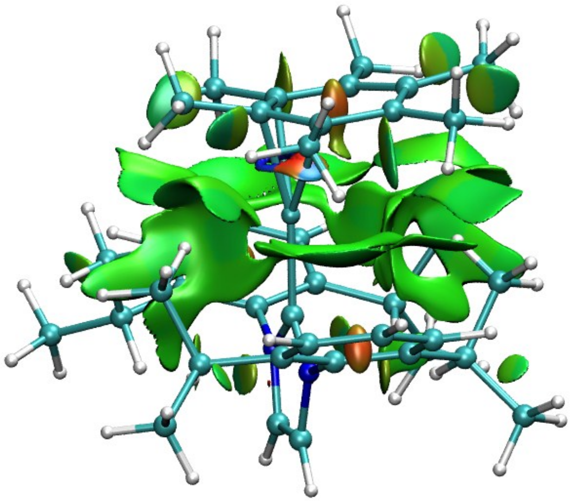

**5**

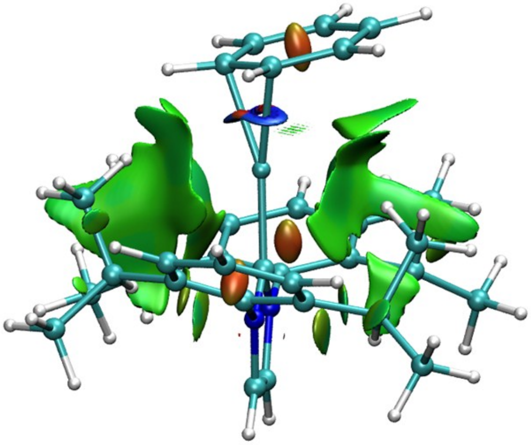

**6**

**S5. Cartesian coordinates** of the four structures (**2**, **3**, **5** and **6**) characterized as real minima of the corresponding potential energy surfaces.

**2**

| | | | |
|---|---|---|---|
| C | -13.839974 | -27.024876 | -3.296973 |
| C | -15.464877 | -28.873660 | -3.260955 |
| C | -16.163156 | -22.185511 | -0.514172 |
| C | -14.680936 | -27.936760 | -3.982614 |
| C | -13.839555 | -26.991307 | -1.877480 |
| C | -12.922086 | -26.143998 | -4.112519 |
| C | -15.444915 | -28.854402 | -1.844053 |
| C | -16.253130 | -19.993251 | 0.497906 |
| C | -14.588062 | -27.955489 | -1.155522 |
| C | -14.922364 | -20.118542 | 0.892667 |
| C | -17.670055 | -24.788060 | 0.645696 |
| C | -16.834505 | -23.276190 | -1.265141 |
| C | -14.828564 | -22.309187 | -0.117682 |
| C | -13.023226 | -25.948640 | -1.145363 |
| C | -14.685487 | -27.935514 | -5.492393 |
| C | -14.641357 | -22.781776 | -3.487757 |
| C | -19.947205 | -22.507053 | -1.988565 |
| C | -16.875389 | -21.023690 | -0.204291 |
| C | -14.211034 | -21.278141 | 0.584149 |
| C | -16.633770 | -21.262100 | -3.788396 |
| C | -14.466926 | -28.087494 | 0.345370 |
| C | -19.269893 | -28.266685 | -2.745239 |
| C | -16.164711 | -22.720580 | -3.688870 |
| C | -18.334103 | -26.171626 | 0.551693 |
| C | -16.262670 | -29.903280 | -4.026300 |
| C | -18.280306 | -26.927791 | -5.394054 |
| C | -16.529597 | -23.466936 | -4.981935 |
| C | -16.287765 | -29.813867 | -1.035776 |
| C | -16.307249 | -24.940186 | 1.340630 |
| C | -21.767038 | -24.890042 | -1.600739 |
| C | -21.304522 | -23.636274 | -4.422743 |
| C | -21.197397 | -26.947090 | -4.674101 |
| C | -18.574205 | -23.833019 | 1.437823 |
| Cu | -16.006426 | -26.693625 | -2.576251 |
| H | -16.809513 | -19.092978 | 0.734532 |
| H | -14.440014 | -19.315339 | 1.438873 |
| H | -14.276490 | -23.211894 | -0.353214 |
| H | -17.908542 | -20.922723 | -0.516325 |
| H | -13.175429 | -21.379509 | 0.890229 |
| H | -11.991250 | -26.276320 | -0.985070 |
| H | -13.448057 | -25.717776 | -0.170783 |
| H | -12.987856 | -25.012577 | -1.700227 |
| H | -13.816898 | -28.473963 | -5.886753 |

| | | | |
|---|---|---|---|
| H | -14.643381 | -26.922229 | -5.892026 |
| H | -15.573444 | -28.405230 | -5.905539 |
| H | -14.332287 | -23.809838 | -3.290868 |
| H | -14.316440 | -22.156444 | -2.656889 |
| H | -14.125408 | -22.434264 | -4.386288 |
| H | -19.214319 | -21.969544 | -2.589778 |
| H | -19.546824 | -22.634771 | -0.984693 |
| H | -20.832945 | -21.868227 | -1.907031 |
| H | -16.430930 | -20.710620 | -2.869835 |
| H | -17.703967 | -21.206454 | -3.996562 |
| H | -16.108156 | -20.758215 | -4.603208 |
| H | -14.321454 | -29.134040 | 0.620790 |
| H | -15.364360 | -27.744654 | 0.866402 |
| H | -13.618858 | -27.538370 | 0.746029 |
| H | -20.046885 | -28.234897 | -1.975069 |
| H | -18.307119 | -28.253467 | -2.232967 |
| H | -19.363519 | -29.225358 | -3.264118 |
| H | -17.687030 | -26.885393 | 0.032763 |
| H | -19.283567 | -26.124735 | 0.014611 |
| H | -18.532618 | -26.561336 | 1.551751 |
| H | -15.639600 | -30.383737 | -4.783081 |
| H | -17.113165 | -29.451399 | -4.541011 |
| H | -16.645298 | -30.694187 | -3.388076 |
| H | -17.230125 | -26.890038 | -5.102370 |
| H | -18.455051 | -26.092612 | -6.079190 |
| H | -18.440765 | -27.849101 | -5.963221 |
| H | -17.610775 | -23.487647 | -5.135453 |
| H | -16.170160 | -24.500262 | -4.958929 |
| H | -16.074571 | -22.972732 | -5.842273 |
| H | -17.185215 | -30.123834 | -1.563575 |
| H | -16.615576 | -29.364804 | -0.098904 |
| H | -15.725050 | -30.718309 | -0.781154 |
| H | -15.858466 | -23.977355 | 1.580723 |
| H | -15.622196 | -25.490408 | 0.693172 |
| H | -16.419197 | -25.495228 | 2.275171 |
| H | -21.338884 | -25.002105 | -0.599507 |
| H | -22.117586 | -25.875743 | -1.914874 |
| H | -22.648647 | -24.248293 | -1.503356 |
| H | -22.097231 | -24.313046 | -4.742768 |
| H | -20.567375 | -23.581508 | -5.229815 |
| H | -21.751090 | -22.641553 | -4.327460 |
| H | -21.339103 | -26.288200 | -5.532304 |
| H | -22.016331 | -26.773809 | -3.974419 |
| H | -21.307055 | -27.973908 | -5.040199 |
| H | -19.569490 | -23.773849 | 0.993005 |
| H | -18.151638 | -22.827685 | 1.477847 |
| H | -18.686374 | -24.188835 | 2.464972 |
| H | -13.471959 | -25.389431 | -4.680267 |
| H | -12.371220 | -26.745682 | -4.838956 |

| | | | |
|---|---|---|---|
| H | -12.182106 | -25.631126 | -3.504006 |
| N | -16.868723 | -23.432600 | -2.595998 |
| N | -17.508310 | -24.316232 | -0.749327 |
| N | -19.174650 | -25.286928 | -3.045548 |
| Si | -17.567996 | -25.144218 | -2.411448 |
| Si | -20.509528 | -24.123088 | -2.779921 |
| Si | -19.471470 | -26.814704 | -3.933269 |

**3**

| | | | |
|---|---|---|---|
| C | -22.855824 | 17.396611 | 1.527478 |
| C | -24.050357 | 22.278252 | 1.877667 |
| C | -23.684904 | 21.992847 | 0.553616 |
| C | -24.675811 | 21.803741 | -0.422206 |
| C | -26.032837 | 21.900874 | -0.074972 |
| C | -26.399383 | 22.183138 | 1.251918 |
| C | -25.406866 | 22.373143 | 2.228481 |
| C | -19.154555 | 18.001061 | 1.095387 |
| C | -22.762904 | 18.434924 | -1.429495 |
| C | -18.664544 | 16.695199 | 1.100673 |
| C | -21.402930 | 17.158077 | 1.357925 |
| C | -24.594151 | 14.682167 | 2.268427 |
| C | -23.182231 | 15.943170 | -1.363114 |
| C | -25.102629 | 17.554235 | -1.452207 |
| C | -20.912726 | 15.849883 | 1.357918 |
| C | -27.233935 | 14.610024 | 0.788081 |
| C | -28.348669 | 18.804500 | 0.542073 |
| C | -29.389878 | 16.986816 | 2.739822 |
| C | -22.997573 | 18.005782 | 4.032057 |
| C | -27.791843 | 19.405491 | 3.549811 |
| C | -27.127896 | 14.834188 | 3.897725 |
| C | -22.455950 | 19.445120 | 4.093447 |
| C | -19.544606 | 15.621631 | 1.231610 |
| C | -21.919958 | 17.010865 | 4.485529 |
| C | -24.222722 | 17.886959 | 4.952025 |
| C | -20.520829 | 18.233697 | 1.227033 |
| C | -23.684739 | 17.320419 | -0.907529 |
| Cu | -25.155966 | 20.314239 | 1.300685 |
| H | -23.286471 | 22.417877 | 2.631922 |
| H | -22.638785 | 21.913215 | 0.285494 |
| H | -24.395489 | 21.583472 | -1.444434 |
| H | -26.797146 | 21.754792 | -0.827424 |
| H | -27.445260 | 22.240407 | 1.523724 |
| H | -25.689323 | 22.580940 | 3.252676 |
| H | -18.472535 | 18.837710 | 0.989518 |
| H | -17.599594 | 16.515282 | 1.003177 |
| H | -21.596564 | 15.016641 | 1.473397 |
| H | -19.166746 | 14.605218 | 1.238563 |
| H | -20.901374 | 19.248906 | 1.212888 |

| | | | |
|---|---|---|---|
| H | -23.085706 | 19.402009 | -1.035187 |
| H | -22.803905 | 18.478342 | -2.520580 |
| H | -21.723976 | 18.273413 | -1.143267 |
| H | -24.056978 | 14.876526 | 1.341060 |
| H | -24.680662 | 13.595987 | 2.375891 |
| H | -23.990541 | 15.043188 | 3.102073 |
| H | -22.175705 | 15.741583 | -0.995550 |
| H | -23.153301 | 15.897896 | -2.454449 |
| H | -23.845752 | 15.150099 | -1.011345 |
| H | -25.808629 | 16.820616 | -1.058447 |
| H | -25.105042 | 17.474632 | -2.540800 |
| H | -25.466352 | 18.551904 | -1.189358 |
| H | -28.235931 | 15.013349 | 0.625183 |
| H | -27.338166 | 13.528050 | 0.918366 |
| H | -26.665173 | 14.769486 | -0.133351 |
| H | -27.546277 | 19.436573 | 0.155795 |
| H | -29.259533 | 19.409687 | 0.589774 |
| H | -28.519226 | 18.012475 | -0.193595 |
| H | -29.549578 | 16.100388 | 2.123792 |
| H | -30.290427 | 17.603339 | 2.644595 |
| H | -29.331324 | 16.669665 | 3.782358 |
| H | -27.675896 | 18.953233 | 4.539525 |
| H | -28.700292 | 20.016000 | 3.577836 |
| H | -26.944129 | 20.076514 | 3.397703 |
| H | -26.924096 | 15.547955 | 4.701518 |
| H | -26.700231 | 13.873908 | 4.202622 |
| H | -28.208496 | 14.698934 | 3.840204 |
| H | -21.589266 | 19.567433 | 3.441637 |
| H | -22.149038 | 19.695613 | 5.111868 |
| H | -23.226896 | 20.154955 | 3.783161 |
| H | -22.260615 | 15.979761 | 4.371462 |
| H | -21.699830 | 17.176088 | 5.542570 |
| H | -20.988862 | 17.127005 | 3.932384 |
| H | -25.007598 | 18.590430 | 4.661285 |
| H | -23.942818 | 18.110870 | 5.983160 |
| H | -24.642260 | 16.879598 | 4.923185 |
| N | -23.799718 | 17.371144 | 0.570170 |
| N | -23.486099 | 17.717096 | 2.664028 |
| N | -26.385978 | 17.161169 | 2.109731 |
| Si | -25.024736 | 18.149990 | 1.724961 |
| Si | -27.932593 | 18.061404 | 2.227649 |
| Si | -26.347863 | 15.376177 | 2.266019 |

**5**

| | | | |
|---|---|---|---|
| C | -10.643982 | -49.947404 | 4.099172 |
| C | -8.210375 | -50.147163 | 3.842611 |
| C | -11.218825 | -52.129967 | 3.870260 |
| C | -9.862199 | -52.052260 | 3.776976 |

| | | | |
|---|---|---|---|
| C | -13.054760 | -50.418393 | 4.135633 |
| C | -7.479546 | -49.997447 | 5.034430 |
| C | -13.671038 | -50.003970 | 2.941568 |
| C | -13.696854 | -50.408430 | 5.388786 |
| C | -8.037491 | -50.425271 | 6.386211 |
| C | -7.738761 | -49.731934 | 2.583736 |
| C | -10.682461 | -46.218278 | 5.321151 |
| C | -6.213835 | -49.404625 | 4.936264 |
| C | -6.470765 | -49.136612 | 2.543252 |
| C | -12.135531 | -45.898449 | 3.350216 |
| C | -14.988412 | -49.532885 | 3.034713 |
| C | -8.563330 | -49.895242 | 1.312046 |
| C | -12.965200 | -50.075438 | 1.592420 |
| C | -9.714244 | -45.704332 | 3.113340 |
| C | -12.967057 | -50.852441 | 6.652960 |
| C | -11.978409 | -46.242735 | 4.717428 |
| C | -5.714804 | -48.977797 | 3.705455 |
| C | -11.010264 | -45.547774 | 2.571622 |
| C | -9.539131 | -46.060412 | 4.475460 |
| C | -15.650354 | -49.494573 | 4.261018 |
| C | -15.013872 | -49.934149 | 5.424154 |
| C | -12.789715 | -48.678942 | 0.973075 |
| C | -13.218564 | -46.467128 | 5.558009 |
| C | -7.103983 | -51.414130 | 7.107790 |
| C | -8.127698 | -46.158919 | 5.014314 |
| C | -10.580136 | -46.183908 | 6.837675 |
| C | -13.880356 | -51.542403 | 7.678658 |
| C | -8.482972 | -45.456472 | 2.269420 |
| C | -7.803457 | -50.690202 | 0.234250 |
| C | -9.021393 | -48.526270 | 0.778263 |
| C | -12.230741 | -49.658050 | 7.292204 |
| C | -13.698471 | -51.028689 | 0.629847 |
| C | -8.336651 | -49.196082 | 7.263604 |
| C | -13.537744 | -45.857052 | 2.786004 |
| C | -11.183545 | -45.019000 | 1.163534 |
| Cu | -10.723950 | -48.091058 | 4.422037 |
| H | -11.892147 | -52.967950 | 3.811822 |
| H | -9.111257 | -52.808463 | 3.625032 |
| H | -8.987872 | -50.936997 | 6.218249 |
| H | -5.616514 | -49.268146 | 5.830081 |
| H | -6.068835 | -48.799204 | 1.594998 |
| H | -15.501233 | -49.201994 | 2.138931 |
| H | -9.463979 | -50.462184 | 1.556973 |
| H | -11.963646 | -50.480829 | 1.748188 |
| H | -12.205452 | -51.581162 | 6.360095 |
| H | -4.733596 | -48.519521 | 3.651523 |
| H | -16.670209 | -49.129797 | 4.311718 |
| H | -15.550387 | -49.906164 | 6.363916 |
| H | -13.753633 | -48.218696 | 0.743297 |

| | | | |
|---|---|---|---|
| H | -12.221610 | -48.743941 | 0.042204 |
| H | -12.255769 | -48.016760 | 1.655704 |
| H | -13.847910 | -45.572786 | 5.575465 |
| H | -12.983452 | -46.724203 | 6.585617 |
| H | -13.824290 | -47.280813 | 5.154984 |
| H | -6.893043 | -52.288393 | 6.487173 |
| H | -7.563972 | -51.760181 | 8.036861 |
| H | -6.148245 | -50.952073 | 7.367245 |
| H | -7.552938 | -46.898779 | 4.455098 |
| H | -8.092849 | -46.465215 | 6.053841 |
| H | -7.600943 | -45.204664 | 4.927810 |
| H | -11.177353 | -45.350826 | 7.214955 |
| H | -9.562746 | -46.029558 | 7.181175 |
| H | -10.951270 | -47.091409 | 7.314443 |
| H | -14.592014 | -50.845656 | 8.128886 |
| H | -13.279278 | -51.956459 | 8.491567 |
| H | -14.447703 | -52.359093 | 7.226669 |
| H | -8.038687 | -44.481577 | 2.498554 |
| H | -8.695415 | -45.480014 | 1.204307 |
| H | -7.714722 | -46.206844 | 2.455891 |
| H | -6.915171 | -50.155501 | -0.110873 |
| H | -8.444639 | -50.857456 | -0.634963 |
| H | -7.480364 | -51.663854 | 0.610583 |
| H | -9.586280 | -47.978657 | 1.535342 |
| H | -9.658004 | -48.650916 | -0.101015 |
| H | -8.166391 | -47.911233 | 0.488612 |
| H | -11.553298 | -49.190800 | 6.575200 |
| H | -11.642336 | -49.980098 | 8.154959 |
| H | -12.943001 | -48.900728 | 7.627842 |
| H | -14.701479 | -50.665635 | 0.391900 |
| H | -13.800486 | -52.028265 | 1.059704 |
| H | -13.147022 | -51.117948 | -0.309739 |
| H | -7.426759 | -48.629510 | 7.478161 |
| H | -8.777424 | -49.499377 | 8.216517 |
| H | -9.038521 | -48.525230 | 6.765074 |
| H | -14.084909 | -44.982539 | 3.154225 |
| H | -14.107008 | -46.738149 | 3.084040 |
| H | -13.554066 | -45.823588 | 1.700764 |
| H | -12.137011 | -44.510659 | 1.035503 |
| H | -11.137887 | -45.822810 | 0.419779 |
| H | -10.408966 | -44.296930 | 0.909524 |
| N | -11.674540 | -50.833816 | 4.070321 |
| N | -9.535593 | -50.710660 | 3.917702 |

**6**

| | | | |
|---|---|---|---|
| C | 6.083847 | 38.826709 | -9.762372 |
| C | 5.261037 | 36.779355 | -10.238556 |
| C | 6.590231 | 36.639245 | -9.995363 |

| | | | |
|---|---|---|---|
| C | 3.669903 | 38.740824 | -10.226428 |
| C | 2.838126 | 38.799344 | -9.095457 |
| C | 1.597752 | 39.423068 | -9.249998 |
| C | 1.214919 | 39.959921 | -10.475714 |
| C | 2.066653 | 39.894418 | -11.572440 |
| C | 3.318609 | 39.280301 | -11.472822 |
| C | 4.293964 | 39.291574 | -12.641767 |
| C | 5.008587 | 40.653740 | -12.686423 |
| C | 3.639262 | 38.961901 | -13.990056 |
| C | 3.298842 | 38.276603 | -7.739967 |
| C | 4.020225 | 39.396283 | -6.967359 |
| C | 2.168695 | 37.670516 | -6.897394 |
| C | 8.432758 | 38.249106 | -9.366800 |
| C | 8.766132 | 38.381644 | -8.010127 |
| C | 10.091407 | 38.706858 | -7.706306 |
| C | 11.028821 | 38.901610 | -8.714191 |
| C | 10.657034 | 38.797704 | -10.051012 |
| C | 9.347098 | 38.470123 | -10.408815 |
| C | 8.914058 | 38.473813 | -11.868021 |
| C | 9.882024 | 37.718858 | -12.789963 |
| C | 8.716690 | 39.929440 | -12.332273 |
| C | 7.737805 | 38.232448 | -6.898300 |
| C | 8.051088 | 37.036833 | -5.985640 |
| C | 7.618992 | 39.535872 | -6.090182 |
| C | 4.659990 | 42.908537 | -8.858580 |
| C | 5.894895 | 42.468935 | -8.335993 |
| C | 7.067795 | 42.602518 | -9.112993 |
| C | 6.995088 | 43.194035 | -10.391363 |
| C | 5.774578 | 43.641136 | -10.880291 |
| C | 4.607261 | 43.493310 | -10.117301 |
| Cu | 6.234753 | 40.659074 | -9.355623 |
| H | 4.505430 | 36.057891 | -10.500185 |
| H | 7.228451 | 35.771828 | -10.002305 |
| H | 0.918422 | 39.487972 | -8.408665 |
| H | 0.243231 | 40.432270 | -10.575720 |
| H | 1.752611 | 40.324638 | -12.516184 |
| H | 5.055619 | 38.527940 | -12.460355 |
| H | 4.029574 | 37.480693 | -7.913096 |
| H | 10.393614 | 38.808161 | -6.669880 |
| H | 12.053558 | 39.148562 | -8.457390 |
| H | 11.394870 | 38.980412 | -10.823501 |
| H | 7.944902 | 37.973621 | -11.943305 |
| H | 6.762500 | 38.050667 | -7.354006 |
| H | 3.761005 | 42.807542 | -8.262113 |
| H | 5.965381 | 42.169086 | -7.295287 |
| H | 8.036610 | 42.389162 | -8.670512 |
| H | 7.898983 | 43.311163 | -10.977645 |
| H | 5.722930 | 44.104262 | -11.859063 |
| H | 3.658633 | 43.841149 | -10.510030 |

| | | | |
|---|---|---|---|
| H | 5.482612 | 40.870413 | -11.727146 |
| H | 4.299574 | 41.460219 | -12.892111 |
| H | 5.782106 | 40.672065 | -13.458007 |
| H | 2.936058 | 39.735922 | -14.307317 |
| H | 3.098777 | 38.013156 | -13.951988 |
| H | 4.402660 | 38.884790 | -14.768469 |
| H | 3.343150 | 40.231043 | -6.766217 |
| H | 4.862259 | 39.779622 | -7.545894 |
| H | 4.402874 | 39.030400 | -6.011537 |
| H | 1.460583 | 38.428094 | -6.552163 |
| H | 2.582532 | 37.191632 | -6.006548 |
| H | 1.609223 | 36.916765 | -7.456302 |
| H | 10.856246 | 38.209600 | -12.855365 |
| H | 9.477021 | 37.671908 | -13.804118 |
| H | 10.046797 | 36.695202 | -12.444917 |
| H | 8.352549 | 39.965079 | -13.362454 |
| H | 9.659681 | 40.481522 | -12.288321 |
| H | 7.993220 | 40.450500 | -11.698332 |
| H | 9.011635 | 37.160669 | -5.478797 |
| H | 8.094564 | 36.103770 | -6.552982 |
| H | 7.282536 | 36.928377 | -5.215354 |
| H | 7.427336 | 40.390595 | -6.746660 |
| H | 8.533467 | 39.752098 | -5.533073 |
| H | 6.798929 | 39.471751 | -5.371524 |
| N | 4.970904 | 38.128008 | -10.087670 |
| N | 7.073473 | 37.907126 | -9.708604 |

**S6**. **Computational details**.

All electronic structure calculations were performed with Gaussian09.[1] We used the B3LYP functional in all cases. The effect of the inclusion of Grimme's dispersion correction term (GD3) was evaluated. We have observed that at the DFT-D level we obtain Cu-Si and Cu-C(carbene) distances shorter than with simple DFT, the former being closer to the experimental ones. For instance, for **2**, the Cu-Si distance is 2.265 Å without dispersion and 2.206 Å with dispersion (experimental = 2.219 Å), and for **6**, the Cu-C distance is 1.908 without dispersion and 1.887 Å with dispersion (experimental = 1.886 Å). On the other side, there is no clear variation of the Cu-arene distance upon inclusion of the dispersion term. For **2**, the shortest Cu-C(arene) distance is 2.298 and 2.311 Å with and without dispersion, respectively, whereas for **6**, they are 2.105 and 2.086 Å with and without dispersion, respectively. It must be remarked that the inclusion of the dispersion term does not modify the hapticities in any case, which are well reproduced at the B3LYP and B3LYP-D3 levels.

The effect of the basis sets on the optimized geometries has been also evaluated. We have concluded that the use of a double-ζ basis set (6-31G*) for all atoms does not reproduce the experimental hapticities, which are obtained only by increasing the size of the basis set at the C and H atoms (6-311+G*). The most relevant bond lengths for both cases can be found in the table below. We have also optimized **6** with the 6-311+G* basis set for all atoms including Cu, but it could not be applied for **2**, **3** and **5** due to computational limitations.

| | 6-31G* all atoms | | | 6-31G* (6-311+G* in C and H) | | | 6-311+G* all atoms | | |
|---|---|---|---|---|---|---|---|---|---|
| Comp. | Hapt. | Cu-Si/C | Cu-C(ar) | Hapt. | Cu-Si/C | Cu-C(ar) | Hapt. | Cu-Si/C | Cu-C(ar) |
| **2** | $\eta^6$ | 2.207 | 2.268 | $\eta^6$ | 2.206 | 2.298 | - | - | - |
| **3** | $\eta^3$ | 2.236 | 2.056 | $\eta^6$ | 2.209 | 2.272 | - | - | - |
| **5** | $\eta^6$ | 1.914 | 2.227 | $\eta^3$ | 1.886 | 2.078 | - | - | - |
| **6** | $\eta^3$ | 1.893 | 2.025 | $\eta^2$ | 1.887 | 2.105 | $\eta^2$ | 1.903 | 2.114 |

After these prospective calculations, all calculations reported in the article were done at the B3LYP-D3 level of theory. The 6-311+G * basis set was employed for C and H and 6-31G* for all other elements. Compounds **2**, **3**, **5** and **6** were characterized as true minima of the PES by vibrational analysis.

The NBO analysis was carried out by using the NBO 3.1 program as implemented in Gaussian09. Complex **6** was decomposed into three fragments (Cu + NHC ligand + $C_6H_6$) for the second order perturbation analysis, while complex **3** were decomposed into five fragments (SiCu + $C_6H_6$ + $C_3H_9Si$ + $C_3H_9Si$ + $C_{15}H_{23}N_2$).

NCI isosurfaces (s = 0.3 au) were obtained from promolecular densities by means of the NCIPLOT program.[2] "Atoms in Molecules" (AIM) analysis was done with the AIMAll software on the B3LYP electron density.[3]

The energy was decomposed by means of the second-generation absolutely localized molecular orbital energy decomposition analysis (ALMO-EDA)[4, 5] implemented in Q-Chem[6] 5.0 at the B3LYP-D3 level of theory with CP correction of the BSSE. The ALMO-EDA decomposes the intermolecular interaction energy into further several interactions, namely a frozen term, a polarization effect term and a charge transfer contribution ($\Delta E_{int} = \Delta E_{FRZ} + \Delta E_{Pol} + \Delta E_{CT}$). Physically, $\Delta E_{FRZ}$ contains three contributions that arise without any relaxation of the fragment

orbitals: attractive dispersion ($\Delta E_{Disp}$), Pauli repulsion ($\Delta E_{Pauli}$) and permanent electrostatics ($\Delta E_{Elec}$).[4]

## S7. REFERENCES:


1. Gaussian 09, Revision D.01, Frisch, M. J.; Trucks, G. W.; Schlegel, H. B.; Scuseria, G. E.; Robb, M. A.; Cheeseman, J. R.; Scalmani, G.; Barone, V.; Mennucci, B.; Petersson, G. A.; Nakatsuji, H.; Caricato, M.; Li, X.; Hratchian, H. P.; Izmaylov, A. F.; Bloino, J.; Zheng, G.; Sonnenberg, J. L.; Hada, M.; Ehara, M.; Toyota, K.; Fukuda, R.; Hasegawa, J.; Ishida, M.; Nakajima, T.; Honda, Y.; Kitao, O.; Nakai, H.; Vreven, T.; Montgomery, J. A., Jr.; Peralta, J. E.; Ogliaro, F.; Bearpark, M.; Heyd, J. J.; Brothers, E.; Kudin, K. N.; Staroverov, V. N.; Kobayashi, R.; Normand, J.; Raghavachari, K.; Rendell, A.; Burant, J. C.; Iyengar, S. S.; Tomasi, J.; Cossi, M.; Rega, N.; Millam, J. M.; Klene, M.; Knox, J. E.; Cross, J. B.; Bakken, V.; Adamo, C.; Jaramillo, J.; Gomperts, R.; Stratmann, R. E.; Yazyev, O.; Austin, A. J.; Cammi, R.; Pomelli, C.; Ochterski, J. W.; Martin, R. L.; Morokuma, K.; Zakrzewski, V. G.; Voth, G. A.; Salvador, P.; Dannenberg, J. J.; Dapprich, S.; Daniels, A. D.; Farkas, Ö.; Foresman, J. B.; Ortiz, J. V.; Cioslowski, J.; Fox, D. J. Gaussian, Inc., Wallingford CT, 2009.
2. J. Contreras-García, E. R. Johnson, S. Keinan, R. Chaudret, J.-P. Piquemal, D. N. Beratan and W. Yang, *J. Chem. Theory Comput.*, 2011, **7**, 625-632.
3. AIMAll (Version 16.08.17), Todd A. Keith, TK Gristmill Software, Overland Park KS, USA, 2016 (aim.tkgristmill.com).
4. P. R. Horn, Y. Mao and M. Head-Gordon, *Phys. Chem. Chem. Phys.*, 2016, **18**, 23067-23079.
5. P. R. Horn and M. Head-Gordon, *J. Chem. Phys.*, 2016, **144**, 084118.
6. Y. Shao, Z. Gan, E. Epifanovsky, A. T. B. Gilbert, M. Wormit, J. Kussmann, A. W. Lange, A. Behn, J. Deng, X. Feng, D. Ghosh, M. Goldey, P. R. Horn, L. D. Jacobson, I. Kaliman, R. Z. Khaliullin, T. Kuś, A. Landau, J. Liu, E. I. Proynov, Y. M. Rhee, R. M. Richard, M. A. Rohrdanz, R. P. Steele, E. J. Sundstrom, H. L. Woodcock, P. M. Zimmerman, D. Zuev, B. Albrecht, E. Alguire, B. Austin, G. J. O. Beran, Y. A. Bernard, E. Berquist, K. Brandhorst, K. B. Bravaya, S. T. Brown, D. Casanova, C.-M. Chang, Y. Chen, S. H. Chien, K. D. Closser, D. L. Crittenden, M. Diedenhofen, R. A. DiStasio, H. Do, A. D. Dutoi, R. G. Edgar, S. Fatehi, L. Fusti-Molnar, A. Ghysels, A. Golubeva-Zadorozhnaya, J. Gomes, M. W. D. Hanson-Heine, P. H. P. Harbach, A. W. Hauser, E. G. Hohenstein, Z. C. Holden, T.-C. Jagau, H. Ji, B. Kaduk, K. Khistyaev, J. Kim, J. Kim, R. A. King, P. Klunzinger, D. Kosenkov, T. Kowalczyk, C. M. Krauter, K. U. Lao, A. D. Laurent, K. V. Lawler, S. V. Levchenko, C. Y. Lin, F. Liu, E. Livshits, R. C. Lochan, A. Luenser, P. Manohar, S. F. Manzer, S.-P. Mao, N. Mardirossian, A. V. Marenich, S. A. Maurer, N. J. Mayhall, E. Neuscamman, C. M. Oana, R. Olivares-Amaya, D. P. O'Neill, J. A. Parkhill, T. M. Perrine, R. Peverati, A. Prociuk, D. R. Rehn, E. Rosta, N. J. Russ, S. M. Sharada, S. Sharma, D. W. Small, A. Sodt, T. Stein, D. Stück, Y.-C. Su, A. J. W. Thom, T. Tsuchimochi, V. Vanovschi, L. Vogt, O. Vydrov, T. Wang, M. A. Watson, J. Wenzel, A. White, C. F. Williams, J. Yang, S. Yeganeh, S. R. Yost, Z.-Q. You, I. Y. Zhang, X. Zhang, Y. Zhao, B. R. Brooks, G. K. L. Chan, D. M. Chipman, C. J. Cramer, W. A. Goddard, M. S. Gordon, W. J. Hehre, A. Klamt, H. F. Schaefer, M. W. Schmidt, C. D. Sherrill, D. G. Truhlar, A. Warshel, X. Xu, A. Aspuru-Guzik, R. Baer, A. T. Bell,

N. A. Besley, J.-D. Chai, A. Dreuw, B. D. Dunietz, T. R. Furlani, S. R. Gwaltney, C.-P. Hsu, Y. Jung, J. Kong, D. S. Lambrecht, W. Liang, C. Ochsenfeld, V. A. Rassolov, L. V. Slipchenko, J. E. Subotnik, T. Van Voorhis, J. M. Herbert, A. I. Krylov, P. M. W. Gill and M. Head-Gordon, *Mol. Phys.*, 2015, **113**, 184-215.
